# Improvement in quality of life among Sri Lankan patients with haemorrhoids after invasive treatment: a longitudinal observational study

**DOI:** 10.1093/bjsopen/zrab014

**Published:** 2021-04-30

**Authors:** S Y J Keong, H K Tan, M D Lamawansa, J C Allen, Z L Low, T Østbye

**Affiliations:** 1 Duke-NUS Medical School, Singapore; 2 Division of Surgery and Surgical Oncology, Singapore General Hospital, Singapore; 3 Department of Surgery, Teaching Hospital Peradeniya, Kandy, Sri Lanka

## Abstract

**Background:**

Haemorrhoids is a common chronic disease that can significantly impact patients’ quality of life. Yet, few studies have evaluated health-related quality of life (HRQoL) of patients with haemorrhoids before and after treatment. This study investigated the HRQoL of patients with haemorrhoids before and after treatment and the change in HRQoL from baseline.

**Methods:**

A prospective observational study of patients with haemorrhoids was conducted at two public hospitals in Kandy, Sri Lanka. Two questionnaires assessing symptom severity and haemorrhoid-specific QoL were administered at initial consultation and at 4- and 8-week follow-ups after treatment (sclerotherapy, rubber band ligation (RBL), haemorrhoidectomy or evacuation of haematoma). The primary outcome was the least squares (LS) change of HRQoL score from baseline, measured using the Short Health Scale adapted for Haemorrhoidal Disease (4 domains: symptom load, interference with daily activities, concern, general well-being).

**Results:**

In 48 patients selected for this study, LS mean change from baseline showed significant improvement in HRQoL across all domains and total Short Health Scale adapted for Haemorrhoidal Disease score at 4- and 8-week follow-ups (*P* < 0.001). Difference in LS mean change from baseline also showed continued improvement of HRQoL from week 4 to week 8 (*P* < 0.010). ‘Concern’ showed greatest improvement at 4 and 8 weeks (*P* < 0.001). Averaged LS mean changes from baseline showed RBL had greater improvement of HRQoL compared with sclerotherapy (*P* = 0.004).

**Conclusion:**

Patients with haemorrhoids had improved HRQoL after invasive treatment. Haemorrhoid-specific QoL is an important component of the extent of disease and can serve as an aid to guide treatment, assess outcomes and monitor disease.

## Introduction

Haemorrhoids is a chronic and recurring disease that can significantly disrupt patients’ daily lives and well-being^1^. It affects about one third of the population^2^ and is most common in adults in their fifties^3^. More than half develop haemorrhoids during their lifetime^4^. Yet, quality of life of patients with haemorrhoids has not been well studied in low-to-middle-income countries (LMICs). Although haemorrhoids is prevalent in Sri Lanka, no studies have been conducted in Sri Lanka to evaluate the health-related quality of life (HRQoL) of patients with haemorrhoids.

The occurrence of symptomatic haemorrhoids does not correlate strongly with higher-grade haemorrhoids^5^. There is also poor correlation between the degree of prolapse and patient symptoms^6^. Therefore, lower-grade haemorrhoids can still be associated with severe symptoms and greatly affect daily life and well-being, which makes the overall effect of haemorrhoids difficult to assess^7^. When considering symptom severity, past research found a significant impact on patients’ quality of life (QoL)^7^. Therefore, using haemorrhoid-specific QoL may better reflect the burden of disease rather than using clinical assessment alone.

Treatment of haemorrhoids ranges from lifestyle modifications to surgical intervention. In Kandy, Sri Lanka, invasive treatment options include sclerotherapy with 5 per cent phenol, rubber band ligation (RBL), haemorrhoidectomy, and evacuation of haematoma if present. Treatment is typically guided by the classification of haemorrhoids^6^^,^^8^. The existing grading of haemorrhoids to guide treatment is not completely satisfactory, however^9^. There is poor correlation between the grade of haemorrhoids and symptoms^6^, so the decision to treat and the type of treatment to recommend must also be guided by the severity of symptoms and their impact on HRQoL.

Standardized and validated outcome measures comparing different treatment options have also been lacking^7^^,^^10^. The Haemorrhoidal Disease Symptom Score (HDSS)^11^ and the Short Health Scale adapted for Haemorrhoidal Disease (SHS_HD_)^10^ have been found to be reliable, responsive and valid as outcome measures^10^. These two scoring systems evaluate symptom severity and haemorrhoid-specific QoL respectively. Using these questionnaires before and after treatment can further assess treatment outcomes of patients with haemorrhoids within the Sri Lankan sociocultural context. With limited resources and high patient load, standardized outcome measures using haemorrhoid-specific QoL may also guide treatment recommendations and inform best practices.

This study aimed to investigate the HRQoL of patients with haemorrhoids in Sri Lanka. The primary objective was to investigate the HRQoL of patients with haemorrhoids before and after invasive treatment (sclerotherapy with 5 per cent phenol, RBL, haemorrhoidectomy or evacuation of haematoma if present) and the change in HRQoL from baseline. The secondary objectives were to investigate the change in HRQoL of patients with haemorrhoids across the different invasive treatments, the severity of symptoms before and after invasive treatment, and the association of potential risk factors with baseline HRQoL scores. Finally, the cultural perceptions of haemorrhoids and the health-seeking behaviours of patients were evaluated.

## Methods

### Study setting and design

A prospective observational study was conducted in Kandy, Sri Lanka, between October 2019 and February 2020. Enrolled patients with haemorrhoids were followed up 4 weeks and 8 weeks after initiation of treatment, or if they received no treatment, after initial consultation. Ethical approval for the study was obtained from the Ethics Review Committee at the University of Peradeniya and the Institutional Review Board at National University of Singapore.

Sample size calculation estimated that 50 patients were required to achieve 80 per cent power at alpha 0.05 to detect a clinically relevant effect size of 0.4 in HRQoL before and after treatment.

### Sampling and selection strategy

Patients were recruited from the general surgical and rectal outpatient clinics and inpatient wards at the Teaching Hospital Peradeniya and Teaching Hospital Kandy, the two largest public hospitals in the Central Province of Sri Lanka. They had to be at least 18 years old and present with haemorrhoids as the primary complaint. Patients might have been newly or previously diagnosed with haemorrhoids but had not received any invasive treatment in the 6 months before recruitment. Convenience sampling was used.

Patients were approached for participation in the study by a Sinhala-speaking research assistant. Exclusion criteria were: active medical conditions associated with pain or bleeding per rectum (e.g., anal fissures or fistula, rectal prolapse), surgical procedures to the anorectal region within the past 6 months or cognitive or language limitations that would affect completion of questionnaires. The diagnosis of haemorrhoids was confirmed by the surgeon during the initial consultation. Written consent was obtained from the participants.

### Data collection, questionnaire content and administration

The HDSS and SHS_HD_ questionnaires (*Appendix S1*), used together, give an overall perspective of symptoms experienced and their impact on daily life and well-being^10^.

The HDSS assessed symptom severity based on the frequency of five cardinal haemorrhoid-related symptoms over the past 3 months – pain, itching, bleeding, soiling and prolapse^10^^,^^11^.

The SHS_HD_ assessed haemorrhoid-specific QoL of patients^10^. It is the only disease-specific QoL tool that has been shown to be reliable, responsive and valid in accordance with the Consensus-based Standards for the selection of health Measurement Instruments (COSMIN) guidelines, especially as a post-treatment outcome measure^10^. Other symptom-based scoring systems for haemorrhoids had not been tested for all three measurement properties.

Both questionnaires were administered by an interviewer in Sinhala at the initial visit (baseline). An additional qualitative survey was also administered to investigate patients’ knowledge and perceptions of haemorrhoids and their health-seeking behaviours. The verbal questions and answers were in Sinhala, but data were recorded in English. There was no interference with regular patient treatment.

The HDSS and SHS_HD_ were re-administered at the 4- and 8-week follow-ups. Upon completion of the 4-week follow-up questionnaires in person, patients were compensated 300 Sri Lankan Rupees for participation and transportation costs. Patients who were unable to attend follow-up at the hospital were followed up via telephone call. All 8-week follow-ups were conducted via telephone call for patients’ convenience. Patients lost to follow-up were not included in the analysis.

Questions on sociodemographics of patients with haemorrhoids including age^3^, sex^3^, BMI^5^, occupation and income level, symptoms common to haemorrhoids (rectal bleeding, perianal pain, itching, soiling, swelling or lump at anus^5^^,^^12^), bathroom habits (constipation^13^^,^^14^, straining^15^) and strenuous activity, such as heavy lifting, were included. Clinical findings and treatment recommendations were recorded after evaluation.

### Variables and co-variables

The primary outcome variable was the change of HRQoL score from baseline. HRQoL was measured using the SHS_HD_^10^ which evaluates four domains: symptom load, interference with daily activities, concerns and general well-being. Each domain was assessed using a 7-point Likert scale. An overall maximum score is 28 points and overall minimum score is 4 points. A lower SHS_HD_ score reflects greater HRQoL.

Patients who underwent invasive treatment received one of the following: sclerotherapy with 5 per cent phenol, RBL, haemorrhoidectomy or evacuation of haematoma if present.

Baseline demographics (age, sex, BMI) and baseline HRQoL scores were considered co-variables as these factors are known to influence self-perceived HRQoL scores.

### Statistical analysis

Continuous variables were summarized as mean(s.d.). Categorical data were summarized using frequencies. Missing height, weight and calculated BMI values were not imputed. Missing income values were accounted for by means imputation.

A repeated measures linear mixed model analysis on change of HRQoL scores from baseline was performed with subjects as random effects, weeks (4 and 8) as fixed effects and baseline HRQoL score as a co-variable, with post-hoc pairwise comparisons. This analysis was used as the study involved repeated measurements on individual patients at three different time points. A similar analysis was performed on change of HDSS scores from baseline.

The above analysis model was augmented to include fixed effect terms for treatment group and week x treatment group interaction to compare the HRQoL of patients with haemorrhoids among the different invasive treatment groups (sclerotherapy, RBL, haemorrhoidectomy). For each week, contrasts were used to test a two degree-of-freedom null hypothesis of no difference among the three treatment groups (H_0_: μ_1_ = μ_2_ = μ_3_). If the null hypothesis was rejected, post-hoc pairwise comparisons were performed. A similar analysis was performed to compare post-treatment HRQoL between patients who had received previous invasive treatment and patients who had not.

The LASSO stepwise algorithm for general linear models (GLM) was used to investigate associations of potential risk factors with baseline HRQoL scores, using significance levels to enter and stay of *P* < 0.050. Optimal selection is based on the Akaike information criteria. Subsequently, a GLM analysis was performed on selected factors to obtain Type III sums of squares and corresponding F-test *P* values. For all tests, *P* < 0.050 was considered statistically significant. Data were analysed using SAS^®^ version 9.4 (SAS Institute, Cary, North Carolina, USA).

## Results

### Patients

**Fig. 1:**
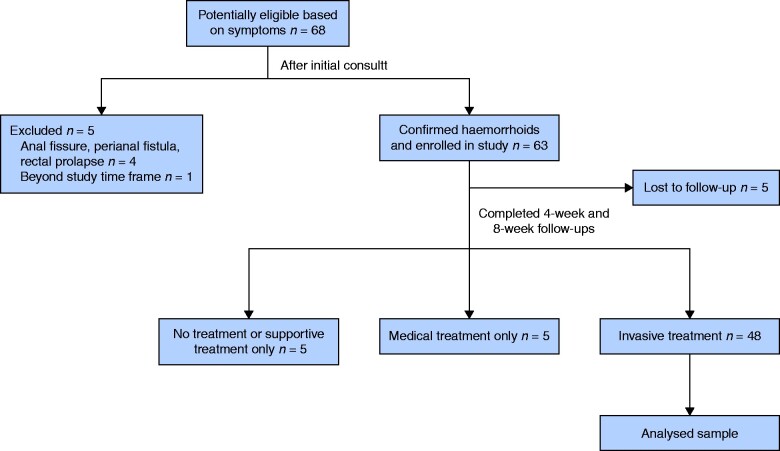
Flow chart for the observational study.

Note: Figure Replacement Requested.

A flow chart of the patients who participated in the study is shown in *Fig. 1*. Fifty-eight patients completed both 4- and 8-week follow-ups. Of these, 48 patients received invasive treatment, five received medical treatment and five received supportive or no treatment. As the number of patients who received medical treatment and supportive or no treatment was small, the analysis was focused on those patients who received invasive treatment (28 males and 20 females, mean age: 47.7 years; *Table 1*).

**Table 1:** Patients’ characteristics at baseline

Variable	No. of Patients (*n* = 48)	Variable	No. of Patients (*n* = 48)
**Demographics**			
Age (years)^*^	47.7(14.3)	BMI (kg/m^2^)^*^	22.5(2.88)
Age categories (years)		BMI categories	
18–34 35–44 45–59 ≥60	9 (19) 8 (17) 23 (48) 8 (17)	Underweight and normal Overweight, pre-obese, obese Unknown	21 (44) 12 (25) 15 (31)
Income per month (1000 Rs)		Occupation	
<25 25 to <50 50 to <75 ≥75	15 (31) 17 (35) 11 (23) 5 (10)	Blue collar White collar Housewife Others^†^	20 (42) 14 (29) 7 (15) 7 (15)
Sex Male Female	28 (58) 20 (42)	Income per month (1000 Rs)^*^	38.5 (28.4)
**Bathroom habits**			
Straining (times per week) <1 1 2 3 4 >4	29 (60) 0 (0) 3 (6) 4 (8) 5 (10) 7 (15)	Bowel movement (per week) <3 ≥3	1 (2) 47 (98)
		Constipation	5 (10)
		Hard stool	21 (44)
**Lifestyle**			
Exercise (h/wk)^*^	0.59 (1.90)	Exercise	9 (19)
Strenuous physical activity (h/wk)^*^	1.92 (7.99)	Strenuous physical activity	6 (13)
Alcohol	6 (13)	Smoking	4 (8)
**Diet**			
Fruits and vegetables (servings/day)^*^	0.77 (1.08)	Water intake (l/day)^*^	1.88 (1.03)
**Personal medical/family history**			
History of haemorrhoids	37 (77)	Family history of haemorrhoids	21 (44)
Years living with haemorrhoids^*^	6.36 (8.63)	Previous treatment for haemorrhoids	23 (48)
**Type of previous treatment for haemorrhoids**			
Traditional/home treatment^‡^	6 (13)	Supportive^§^	4 (8)
Medical^¶^	5 (10)	Invasive^#^	15 (31)
**Females only (*n* = 20)**			
Pregnant^**^	0/20 (0)	Number previous pregnancies 0 1 2 3 ≥4	1 (5) 2 (10) 9 (45) 3 (15) 5 (25)

Values in parentheses are percentages unless indicated otherwise; *values are mean(s.d.).

† Retirees, students and unemployed.

‡ Ayurvedic medicine and home remedies. ^§^Diet and lifestyle changes, increase water intake, reduce straining, Sitz bath.

¶ Lactulose, lignocaine gel or ibuprofen.

# Sclerotherapy, rubber band ligation, haemorrhoidectomy or evacuation of haematoma.

** Currently or recently pregnant within the past 6 months from time of recruitment.

Thirteen patients had missing self-reported height, and five patients had missing self-reported weight, resulting in missing/unknown BMI data, while four patients had missing income values. Thirty-seven patients had a history of haemorrhoids. Fifteen patients had previously tried invasive treatment for haemorrhoids (*Table 1*).

The most common complaints were swelling/prolapse (36 patients) and rectal bleeding (33 patients). Swelling/prolapse (HDSS) was the symptom most commonly rated as most severe. Forty-six patients had internal haemorrhoids only. One patient had both internal and external haemorrhoids, and one patient had external haemorrhoids only. Twenty-five patients were treated with sclerotherapy, while seventeen patients had RBL (*Table 2*). Other patient characteristics, symptoms and clinical features at baseline are presented in *Tables 1* and *2*.

**Table 2:** Reported symptoms, clinical findings, treatment prescribed and symptom severity at baseline

Variable	No. of Patients (*n* = 48)
**Reported Symptoms**	
Rectal bleeding Itching Swelling/prolapse	33 (69) 11 (23) 36 (75)
Perianal pain Soiling	31 (65) 12 (25)
**Clinical findings and type of invasive treatment prescribed**	
External haemorrhoids^†^	2 (4)
Internal haemorrhoids^†^	47 (98)
Grade of internal haemorrhoids 0^‡^ 1 2 3 4	1 (2) 2 (4) 33 (69) 11 (23) 1 (2)
Position of internal haemorrhoids^§^ 3 o’clock 6 o’clock 7 o’clock 8 o’clock 9 o’clock 11 o’clock	37 (79) 1 (2) 33 (70) 2 (4) 1 (2) 33 (70)
Thrombosed haemorrhoid	2 (4)
Invasive treatment prescribed Evacuation of haematoma Sclerotherapy Rubber band ligation Haemorrhoidectomy	2 (4) 25 (52) 17 (35) 4 (8)
**Symptom severity (HDSS)^¶^**	
Pain*	2.15(1.74)
Pain score 0 1 2 3 4	17 (35) 1 (2) 4 (8) 10 (21) 16 (33)
Bleeding*	1.67(1.58)
Bleeding score 0 1 2 3 4	18 (38) 6 (13) 7 (15) 8 (17) 9 (19)
Swelling/prolapse*	3.00(1.68)
Swelling/prolapse score 0 1 2 3 4	10 (21) 2 (4) 0 (0) 2 (4) 34 (71)
Itching*	0.77(1.42)
Itching score 0 1 2 3 4	36 (75) 1 (2) 1 (2) 6 (13) 4 (8)
Soiling*	0.83(1.49)
Soiling score 0 1 2 3 4	35 (73) 2 (4) 1 (2) 4 (8) 6 (13)
Total HDSS score*	8.42(4.17)
Total HDSS score category 0–5 6–10 11–15 16–20	17 (35) 16 (33) 12 (25) 3 (6)

Values in parentheses are percentages unless indicated otherwise;

* values are mean(s.d.).

† External and internal haemorrhoids are not mutually exclusive.

‡ Indicates no internal haemorrhoids.

§ Numbers and percentages reflect multiple haemorrhoids per subject, so do not sum to total and/or 100%.

**^¶^** Haemorrhoidal Disease Symptom Score (HDSS) assesses symptom severity based on frequency of symptoms (0, never; 1, less than once a month; 2, less than once a week; 3, 1–6 days a week; 4, every day). The larger the HDSS score, the more severe the symptoms.

### Health-related quality of life scores

Baseline HRQoL scores were significantly associated with post-treatment HRQoL scores and with change from baseline for all SHS_HD_ domains and total SHS_HD_ score (*P* < 0.001). Patients with higher baseline HRQoL scores (poorer HRQoL) had lower post-treatment HRQoL scores (better HRQoL) and greater change from baseline HRQoL scores (greater improvement in HRQoL). Thus, baseline HRQoL was included as a co-variable in the analysis model. Age, sex and BMI had no significant effect on HRQoL scores or change from baseline and were therefore excluded.

Post-treatment adjusted least squares (LS) mean scores showed significant differences between weeks 4 and 8 (*P* < 0.010). Lower SHS_HD_ score reflects greater HRQoL. Positive values for LS mean change from baseline indicate improved HRQoL. LS mean change from baseline showed significant improvement in HRQoL across all domains and total SHS_HD_ score at week 4 and week 8 (*P* < 0.001). Differences in LS mean change from baseline were negative, indicating that HRQoL scores continued to improve from week 4 to week 8 (*P* < 0.010). Of the HRQoL domains, concern had the largest mean score at baseline, suggesting patients were most worried about their symptoms compared with other domains of the SHS_HD_. Notably, concern also showed greatest improvement at weeks 4 and 8 and from week 4 to week 8 (*Table 3*).

**Table 3:** Health-related quality of life (HRQoL) least squares mean scores at baseline and at weeks 4 and 8 after invasive treatment, and difference in change from baseline

SHS_HD_ domain	**Mean(s.d.) score**	LS mean(s.d.) score^*^^†^		*P* (F-test)	LS mean change from BL (95% c.i.)		Difference in LS mean change from BL^‡^**(95**% c.i.)
	BL	W4	W8		ΔBL W4	ΔBL W8	ΔBL W4 – ΔBL W8
**Symptom load**	3.08(1.35)	1.79(0.89)	1.56(0.87)	0.002	1.29^§^^#^ (1.03, 1.55)	1.52^#^ (1.27, 1.77)	−0.23^¶^ (-0.37, -0.09)
**Interference**	2.73(1.83)	1.63(0.91)	1.35(0.89)	0.002	1.10^#^ (0.84, 1.37)	1.38^#^ (1.12, 1.63)	−0.27^¶^ (-0.44, -0.10)
**Concern**	3.46(1.61)	1.92(1.28)	1.46(0.97)	<0.001	1.54^#^ (1.17, 1.91)	2.00^#^ (1.72, 2.28)	−0.46^#^ (-0.69, -0.23)
**General well-being**	2.35(1.00)	1.85(0.50)	1.58(0.59)	0.001	0.50^#^ (0.35, 0.65)	0.77^#^ (0.60, 0.94)	−0.27^¶^ (-0.43, -0.12)
**Total SHS_HD_ score**	11.6(4.35)	7.19(2.99)	5.96(2.96)	<0.001	4.44^#^ (3.57, 5.31)	5.67^#^ (4.81, 6.52)	−1.23^#^ (-1.67, -0.79)

Adjustment for baseline HRQoL scores was significant at *P* < 0.001 for all domains and total Short Health Scale adapted for Haemorrhoidal Disease (SHS_HD_) score.

* A lower SHS_HD_ domain score and total SHS_HD_ score reflects greater HRQoL.

† Standard deviation estimate from unstructured variance–co-variance matrix.

‡ A negative difference in change from baseline at week 4 (ΔBL W4) – change from baseline at week 8 (ΔBL W8) indicates an improvement from week 4 (W4) to week 8 (W8).

§ Least square differences (LSD) t-test in the context of ANCOVA:

¶ *P* < 0.01;

# *P* < 0.001. LS mean, Least squares mean; BL, baseline.

LS mean change of HRQoL scores (total SHS_HD_ score) from baseline for sclerotherapy, RBL, and haemorrhoidectomy respectively was 3.57, 5.48 and 4.66 at week 4, and 5.01, 6.54 and 5.91 at week 8. Post-hoc comparisons of the different invasive treatments showed a significant difference in change from baseline at week 4 for RBL *versus* sclerotherapy (change from baseline at week 4 (ΔBL W4) = 1.92, *P* = 0.021) but no significant difference at week 8 (ΔBL W8 = 1.54, *P* = 0.062). However, when averaged across weeks 4 and 8, patients who received RBL had greater improvement in HRQoL than those with sclerotherapy (β = 1.73, *P* = 0.004). Averaged differences between haemorrhoidectomy and RBL (*P* = 0.469), and haemorrhoidectomy and sclerotherapy (*P* = 0.306) were not statistically significant. Evacuation of haematoma was not included in this analysis due to small sample size (2 patients).

LS mean change of HRQoL scores (total SHS_HD_ score) from baseline for patients who previously received invasive treatment and those who had not was 3.60 and 4.67 respectively at week 4, and 5.34 and 5.44 at week 8. Averaged differences showed no significant difference between patients who had previously received invasive treatment and those who had not (*P* = 0.430).

### Symptom severity scores

LS mean change of HDSS scores from baseline showed significant improvement in symptom severity across total HDSS score and all domains at weeks 4 and 8 (*P* < 0.050). Bleeding, soiling and total HDSS score had significant differences in change from baseline (*P* < 0.050), indicating that symptom severity continued to improve from week 4 to week 8 in these domains. Improvement in symptom severity for pain, itching, and swelling/prolapse mainly occurred from baseline to week 4 as the differences in change from baseline were not statistically significant (*Table 4*).

**Table 4:** Haemorrhoidal Disease Symptom Score least squares mean scores at baseline and at weeks 4 and 8 after invasive treatment, and difference in change from baseline (adjusted for baseline HDSS scores)

HDSS Domain	Mean(s.d.) score	LS mean(s.d.) score* ^†^		*P* (F-test)	LS Mean Change from BL (95% c.i.)		**Difference in LS Mean Change from BL** ^‡^ **(95% c.i.)**
	BL	W4	W8		ΔBL W4	ΔBL W8	ΔBL W4 – ΔBL W8
**Pain**	2.15 (1.74)	1.00 (1.43)	0.56 (1.21)	0.057	1.15^#^^§^ (0.73, 1.56)	1.58^#^ (1.23, 1.94)	−0.44 (-0.89, 0.01)
**Itching**	0.77 (1.42)	0.48 (0.95)	0.40 (1.03)	0.420	0.29^¶^ (0.01, 0.57)	0.38^¶^ (0.08, 0.67)	−0.08 (-0.29, 0.12)
**Bleeding**	1.67 (1.58)	0.65 (1.08)	0.23 (0.83)	0.034	1.02^#^ (0.71, 1.34)	1.44^#^ (1.20, 1.68)	−0.42^¶^ (-0.80, -0.03)
**Soiling**	0.83 (1.49)	0.38 (1.58)	0.00 (N.E.^**^)	0.114	0.46^¶^ (0.10, 0.82)	0.83^#^ (0.83, 0.83)	−0.38^¶^ (-0.74, -0.01)
**Swelling/prolapse**	3.00 (1.68)	1.42 (1.78)	1.46 (1.80)	0.806	1.58^#^ (1.07, 2.10)	1.54^#^ (1.02, 2.07)	0.04 (-0.30, 0.38)
**Total HDSS Score**	8.42 (4.17)	3.85 (4.10)	2.44 (3.19)	<0.001	4.56^#^ (3.37, 5.75)	5.98^#^ (5.05, 6.90)	−1.42^#^ (-2.13, -0.70)

* Higher Haemorrhoidal Disease Symptom Score (HDSS) domain score and total HDSS score reflect more severe symptoms.

† Standard deviation estimate from unstructured variance–co-variance matrix.

‡ A negative difference in change from baseline at week 4 (ΔBL W4) – change from baseline at week 8 (ΔBL W8) indicates an improvement from week 4 (W4) to week 8 (W8).

§ Least square differences (LSD) t-test in the context of ANCOVA: ^¶^*P* < 0.050; ^#^*P* < 0.001. LS mean, least squares mean; BL, baseline. **Not estimable.

LS mean change of HDSS scores (total HDSS score) from baseline for sclerotherapy, RBL, and haemorrhoidectomy was 3.70, 6.44 and 2.29 respectively at week 4, and 4.98, 7.61 and 5.54 at week 8. Patients who received RBL had greater improvement in symptom severity at week 4 and week 8 compared to those who received sclerotherapy (ΔBL W4 = 2.74, *P* = 0.015; ΔBL W8 = 2.64, *P* = 0.019).

### Risk factors associated with baseline HRQoL score

Higher HDSS scores indicate more severe symptoms. Higher baseline total SHS_HD_ and domain scores reflect poorer baseline HRQoL. Soiling was positively correlated (*P* < 0.010) with total SHS_HD_ score, symptom load, interference and concern. Higher pain scores and eating more fruits and vegetables were also associated with higher baseline concern scores (*Table 5*). Other potential risk factors such as age, sex, BMI, straining, bleeding (HDSS) and swelling/prolapse (HDSS) were not selected in the stepwise regression.

**Table 5:** Risk factors associated with baseline health-related quality of life scores

Selected risk factors	Model coefficient *P*				
	Total SHS_HD_ * score	SHS_HD_ domain			
		Symptom load	Interference	Concern	General well-being
Model R-square	0.27	0.22	0.19	0.32	0.18
Intercept	10.4 **<0.001**	2.73 **<0.001**	2.28 **<0.001**	2.08 **<0.001**	2.13 **<0.001**
**HDSS** ^†^					
Pain				0.27 **0.034**	
Soiling	1.52 **<0.001**	0.43 **0.001**	0.53 **0.002**	0.38 **0.008**	
Itching					0.30 **0.003**
**Diet**					
Fruits and vegetables				0.62 **0.003**	

In addition to the risk factors in the table, age, sex, BMI, straining, bleeding (Haemorrhoidal Disease Symptom Score, HDSS), and swelling/prolapse (HDSS) were also investigated as possible risk factors. *Higher baseline total Short Health Scale adapted for Haemorrhoidal Disease (SHS_HD_) score and domain score reflect poorer baseline health-related quality of life.

† HDSS assesses symptom severity based on frequency of symptoms (0, never; 1, less than once a month; 2, less than once a week; 3, 1–6 days a week; 4, every day). The larger the HDSS score, the more severe the symptoms.

### Perceptions and health-seeking behaviours

In the supplementary qualitative analysis, 30 patients did not know anything about haemorrhoids prior to the initial consultation. Seventeen patients had not seen a doctor for haemorrhoids previously and most gave the reason of not seeing a doctor because they had mild symptoms or symptoms only recently developed. Perianal pain (20 patients) and rectal bleeding (16 patients) were the most common reasons reported by patients for deciding to seek medical care. All 48 patients reported ‘Doctor prescribed’ as the reason for undergoing invasive treatment after the initial consultation.

## Discussion

Haemorrhoids is a benign but common chronic disease, which can disrupt patients’ lives by impacting their daily lives and well-being^1^. As 66 per cent of all employed people in Sri Lanka work in the informal sector, consisting mostly of labour-intensive jobs and dependence on daily salary^16^, haemorrhoids can impact their HRQoL and ultimately affect their livelihoods. Therefore, evaluating HRQoL is imperative in assessing impact of disease, with the goal of treatment to address symptoms^17^ and improve haemorrhoid-specific QoL. Parallel to HRQoL scores, symptom severity also improved across total HDSS score and all domains at 4  and 8 weeks after invasive treatment. As the SHS_HD_ was shown to be responsive and highly correlated with symptom load and patient postoperative satisfaction^10^, improved HRQoL after invasive treatment further emphasizes the benefit of haemorrhoid-specific QoL as a way to assess treatment outcomes together with symptom severity and clinical findings.

Haemorrhoid-specific QoL and overall symptom severity also continued to improve from week 4 to week 8, which may reflect the continued effects of invasive treatments. Most patients did not report taking other traditional or prescribed medication during this period, and none had returned to the hospital for worsening of symptoms or additional treatment. Proctoscopy is not routinely performed to evaluate for resolution of haemorrhoids if symptoms have not worsened. However, as a multisymptomatic disease, resolution of one symptom may not improve HRQoL. Patients’ symptoms may also reduce in frequency but still have a severe impact on their well-being^10^. Improvement in certain symptoms may also contribute more to the improvement in HRQoL. Bleeding and soiling showed significant improvement in symptom severity from week 4 to week 8, whereas pain, itching and swelling/prolapse did not. This highlights the importance of assessing all symptoms of haemorrhoids together with HRQoL. With a high patient load and limited resources in LMICs like Sri Lanka, surgeons may also use the SHS_HD_ and HDSS together as a quick and more robust means to monitor disease recurrence.

Concern in the SHS_HD_ assessed the frequency of haemorrhoid-related worries patients had^10^. Prior to treatment, patients were most concerned about their symptoms out of all SHS_HD_ domains. This emphasizes the psychological impact the disease has on patients, where clinical grading of haemorrhoids alone does not adequately evaluate the whole disease^6^^,^^7^. Concern also had the greatest improvement after invasive treatment at each follow-up, further reflecting that treatment not only addresses the disease but disease-related concerns. As patients appeared to wholly trust surgeons’ recommendations, assessing haemorrhoid-specific QoL may also bring forth their concerns, engage them in the decision-making process and improve patient-centric care.

Higher pain and soiling HDSS scores were associated with greater concern on the SHS_HD_. This differs from other experiences^7^ where frequency of soiling was not significant in a multivariable regression model for impact on QoL. Another author^10^ found that all HDSS domains had a positive correlation with symptom load on the SHS_HD_, whereas in the model in the present study, only soiling showed a positive correlation with symptom load. However, this may be due to the smaller sample. The use of fruits and vegetables was also positively associated with concern, which may reflect patients’ health-seeking behaviours: indeed, patients who were greatly concerned may be more likely to consume fruits and vegetables to alleviate their symptoms.

Patients who received RBL had greater improvement in symptom severity and haemorrhoid-specific QoL after treatment compared to sclerotherapy, similar to other studies that showed RBL to be superior to sclerotherapy in patient-perceived response to treatment^18^. In common practice, sclerotherapy is used to treat grade I and II haemorrhoids while RBL is used for grade II and III haemorrhoids^19^. However, RBL is not available at the Teaching Hospital Peradeniya. As such, sclerotherapy is also used to treat grade III haemorrhoids at this hospital. Future studies could use a larger patient sample to investigate the improvement in symptom severity and haemorrhoid-specific QoL amongst the different types of invasive treatments over a longer period, especially since sclerotherapy and RBL can be considered as a course of treatment (i.e., can be given multiple times)^17^. This may have greater implications in reorganizing medical resources to make RBL available at all public hospitals in Sri Lanka.

As symptom severity has been shown to be related to type of treatment, i.e., ambulatory or operative care^7^^,^^10^, baseline HRQoL could also be assessed in future studies to decide between different types of invasive treatment. The SHS_HD_ is not diagnostic or prognostic^10^, but it serves as an aid, along with clinical assessment, for surgeons when recommending treatment. Public hospitals in Sri Lanka have limited resources and conservative management, such as the use of flavonoids, is not readily available. As such, invasive treatment is the mainstay of treatment of haemorrhoids. Therefore, careful evaluation of HRQoL to guide treatment may prove beneficial.

This study illustrates that haemorrhoid-specific QoL is an important dimension of the impact of the disease on patients, and can serve as an aid for surgeons to guide treatment, assess outcomes and monitor disease. The longitudinal cohort study assessing prospective data is a strength, with fully completed questionnaires at baseline, 4- and 8-week follow-ups. Limitations of the study include the use of convenience sampling and small sample size, restricting generalizability of the results. The SHS_HD_ is a reliable and responsive HRQoL measure in the Danish population^10^. Although the SHS_HD_ had not yet been validated in Sinhala in the Sri Lankan population, the questionnaires were translated by a Sri Lankan healthcare professional and reviewed by a sworn translator. Additionally, the SHS_HD_ is the first haemorrhoid-specific QoL tool^10^, which was adapted from the Short Health Scale, a validated HRQoL measure for patients with inflammatory bowel disease. Future research could cross-validate the SHS_HD_ and further investigate its use in Sinhala in the Sri Lankan population or in other languages to assess haemorrhoid-specific QoL in other LMICs. Further studies could also test the SHS_HD_ against other HRQoL tools to measure criterion validity.

## Supplementary Material

zrab014_Supplementary_Data
