# Supplementary material for: Improvement in quality of life among Sri Lankan patients with haemorrhoids after invasive treatment: a longitudinal observational study
Source: BJS Open. 2021 Apr 30;5(2):zrab014. doi: 10.1093/bjsopen/zrab014 (PMC8088290; doi:10.1093/bjsopen/zrab014)
Supplement: zrab014_Supplementary_Data [file zrab014_supplementary_data.docx]

**Appendix**

Appendix 1. HDSS and SHS_HD_ Questionnaires used at Initial consult, Week 4 and Week 8 after Invasive Treatment.

**Hemorrhoidal Disease Symptom Score (HDSS)^1, 2^**

The following questions deal with **symptoms** caused by hemorrhoids. Your answers should reflect your symptoms **during the last 3 months**. Please only choose **one answer** per question.

Q1. How often do you feel pain from your hemorrhoids?

 Never

 Less than once a month

 Less than once a week

 1-6 days per week

 Every day (always)

Q2. How often do you feel itching or discomfort of the anus?

 Never

 Less than once a month

 Less than once a week

 1-6 days per week

 Every day (always)

Q3. How often do you bleed when passing stool?

 Never

 Less than once a month

 Less than once a week

 1-6 days per week

 Every day (always)

Q4. How often do you soil your underwear (soiling from the anus)?

 Never

 Less than once a month

 Less than once a week

 1-6 days per week

 Every day (always)

Q5. How often do you feel a swelling or a prolapsing hemorrhoid?

 Never

 Less than once a month

 Less than once a week

 1-6 days per week

 Every day (always)

**Short Health Scale_HD_ (SHS_HD_)^1^**

The following questions deal with how your symptoms caused by hemorrhoids affect your daily life. Please only circle **one answer** per question.

Q1. In your view, how severe are your symptoms caused by hemorrhoids? Please grade your symptoms on a 7-point scale, where 1 is “no symptoms” and 7 is “severe symptoms.”

1 2 3 4 5 6 7

No symptoms Severe symptoms

Q2. Do your symptoms interfere with your daily activities? Please grade your symptoms on a 7-point scale, where 1 is “not at all” and 7 is “interfere to a very high degree.”

1 2 3 4 5 6 7

Not at all Interfere to a very high degree

Q3. Do your symptoms cause much concern? Please grade your symptoms on a 7-point scale, where 1 is “no concerns” and 7 is “constant concerns.”

1 2 3 4 5 6 7

No concerns Constant concerns

Q4. How is your general feeling of well-being? Please grade your symptoms on a 7-point scale, where 1 is “very good” and 7 is “very bad.”

1 2 3 4 5 6 7

Very good Very bad

1. The Haemorrhoidal Disease Symptom Score (HDSS) and Short Health Scale_HD_ (SHS_HD_) is taken from Rørvik HD, Styr K, Ilum L, Mckinstry GL, Dragesund T, Campos AH, et al. Hemorrhoidal Disease Symptom Score and Short Health ScaleHD. Diseases of the Colon & Rectum. 2019;62(3):333–42.
2. The HDSS was first proposed by Nyström P-O, Qvist N, Raahave D, Lindsey I, Mortensen N. Randomized clinical trial of symptom control after stapled anopexy or diathermy excision for haemorrhoid prolapse. British Journal of Surgery 2010;97:167-76.
